# Fever therapy revisited

**DOI:** 10.1038/sj.bjc.6602386

**Published:** 2005-02-08

**Authors:** U Hobohm

**Affiliations:** 1 University of Applied Sciences, Bioinformatics, Wiesenstrasse 14, D-35390 Giessen, Germany

**Keywords:** fever therapy, neoplasms, spontaneous neoplasm regression, tumour, oncology

## Abstract

The phenomenon of spontaneous regression and remission from cancer has been observed by many physicians and was described in hundreds of publications. However, suggestive clues on cause or trigger are sparse and not substantiated by much experimental evidence. In this review, literature is surveyed and summarised and possible causes are discussed. At least in a larger fraction of cases a hefty feverish infection is linked with spontaneous regression in time and is investigated as putative trigger. Epidemiological and immunological evidence is put into perspective. An online forum to discuss the possible application of fever therapy in the future can be accessed at http://bioinfo.tg.fh-giessen.d e/fever-and-cancer.

Spontaneous regressions and remissions from cancer have been recognised since the diagnosis of cancer became a science. Rohdenburg, a medical director at Columbia University, wrote in 1918: ‘It can be definitely asserted that regressive changes varying from a temporary standstill to the complete disappearance of the tumour, whether it be of epithelial or connective tissue origin, may occur at any age period, in either sex, and irrespective of the location of the growth’. On the other hand, large fractions of clinicians over large fractions of the century disregarded the phenomenon or denied that its existence has been proven beyond doubt. Although even a quick glance into the literature must convince us that unexplained healings from cancer happen, it is a rare event and, most importantly, the phenomenon of spontaneous healing in cancer remains almost without clue. Here I survey literature on spontaneous regression and remission and try to align with contemporary immunology and epidemiology.

## DISCUSSION

### Spontaneous regressions

The literature on spontaneous regression and remission consists of numerous case studies and few reviews (Rohdenburg, 1918; Boyd, 1966; Everson and Cole, 1968; Stephenson *et al*, 1971; Challis and Stam, 1990). Two of these reviews appeared as books (Boyd, 1966; Everson and Cole, 1968). The reviews of Stephenson *et al* (1971) and of Challis and Stam (1990) are of particular importance, since they were published in scientific journals and passed peer review. Stephenson *et al* were the first to use a computer for statistical data analysis on spontaneous regression. They included the cases mentioned by Everson and Cole and added 48 case reports to a final number of 224 cases (Stephenson *et al*, 1971). Challis and Stam re-reviewed 61 cases mentioned in Boyd (1966), re-reviewed 176 cases mentioned in Everson and Cole (1968) and added 265 articles covering 504 case studies found by manual and computer searches for the period 1966–1987 (mean 24 cases per year).

For the subsequent period 1987–2003, a PubMed query on case reports of spontaneous regressions/remissions/resolutions delivers 136 hits (Query, 2004) (mean 10 reports per year). Since some of the case reports covered more than one patient, we can state a small but ongoing rate of about 1–2 dozen reported cases per year over the past 40 years, with perhaps decreasing tendency.

Regressions and remissions were not always permanent; however, in a considerable number of cases they were long lasting or even cures. According to the Everson and Coles study, in 57/176 (33%) cases the regression lasted more than 5 years; the extended study of Challis and Stam reports 25% patients alive after 5 years, where this is a lower margin, since for 20% of patients no such record could be obtained. Hence, spontaneous regressions can save lives.

The important work of Challis and Stam covering 741 selected cases from the period 1900–1987 has not fuelled much discussion. Presumably a melange of reasons is accountable, namely misdiagnosis, rareness, denial and unexplainability.

First, malign neoplasms were sometimes misdiagnosed, with higher likelihood for reports from the early 20th century. Attempts to avoid false-positive reports (non-neoplasm reported as neoplasm) increases the risk of introducing false-negatives (filtering away true malign neoplasms as misdiagnosis); yet, the authors attempted to be on the safe side. Everson and Cole applied rigorous standards emphasizing clear histologic confirmation, which led them to drop a majority of case reports from consideration. Also, lymphomas and leukaemias were excluded because of the natural changes in growth rate these malignancies undergo, and retinoblastomas were excluded because histological evidence was rarely available. Chalis and Stam again emphasised clear histological confirmation, but included lymphoma and retinoblastoma.

Second, rareness: Omitting to read the case reports but looking at numbers only – less than 1000 published cases in a century in which cancer is among the four main causes of death in industrialised nations – might cast doubt whether spontaneous regression is of any significance with respect to research, even if we assume a large number of unreported cases. A large fraction of unknown cases must be assumed for obvious reasons. Cases will not be observed when the patients after improvement of their condition did not show up in the clinic any more. Observed cases will not be reported, when the responsible physician (i) did not consider the case relevant, (ii) was unable to make sense out of it, (iii) could not rule out effective prior treatment, (iv) was not literate enough or did not take the effort to write a report. An estimation of the rate of spontaneous remission can only be approximate due to lack of solid data. If, for a very coarse estimate, one assumes 50 new cancer cases in a population of 1000 per year, a collective average population size of 600 million people in the US, Europe and Japan and a 100-fold underestimate of spontaneous regression given by the number of published cases (653 in the period 1966–2004), only about 1–10 spontaneous remissions per 1 million cancer cases can be assumed. Yet, exceptional cases can be instructive. In HIV research, long-term nonprogressors (LTNP) – infected people who do not progress into full AIDS for long periods – are a small but interesting cohort of patients. One such cohort consists of individuals with a mutation in both alleles for the CCR5 receptor. CCR5 was identified as one of the receptors used by the virus for entry and is now target of intense research on entry inhibitors, a novel class of drugs (Vermeire and Schols, 2003).

Third, rebuffal. We have to admit that over a large portion of the century a large fraction of clinical oncologists would simply deny any healing capacity of the human body with respect to cancer. Professor Bauer, one of the founders of the German Cancer Research Institute (DKFZ) in Heidelberg, in his founding talk for the institute in 1965 claimed that ‘the human body has no cancer fighting capabilities’. Such a preoccupation cannot be apt to gain scientific insight.

Fourth, unexplainability. Everson and Cole in 1968, in their summary, offered as explanation for spontaneous regression the following: ‘In many of the collected cases […] it must be acknowledged that the factors or mechanisms responsible for spontaneous regression are obscure or unknown in the light of present knowledge. However, in some of the cases, available knowledge permits one to infer that hormonal influences probably were important […]. In other cases, the protocols strongly suggest that an immune mechanism was responsible […]’. Challis and Stam in 1989, even more at a loss, survey ‘In summary, we are left to conclude that, although a great number of interesting and unusual cases continue to be published annually, there is still little conclusive data that explains the occurrence of spontaneous regression.’ This lack of understanding persists until today, as shown by an arbitrarily chosen example published in a high impact journal. In an article in the Proceedings of the National Academy of Sciences, the authors wrote in 2003 (Cui *et al*, 2003): ‘Despite efforts over many decades, the mechanism(s) of spontaneous regression in humans and animals has remained elusive.’ Apparently, 20th century medicine provided perplexingly few explanations facing this intriguing phenomenon. Yet, an unusually strong immune reaction might play a role.

### Tumour immunology

Cancer patients usually carry tumour-reactive T-cells, that is, T-cells that recognise tumour cell antigens. It was known early on not only that tumours are invaded by defence cells but also that the number of invading cells and survival rate can correlate (Black *et al*, 1956). This exciting observation published in 1956, however, did not lead into any research activity aimed at strengthening the defence capacities of the body, but rather was overshadowed by hopes and hypes offered by the emerging chemotherapy. The first molecular identification of tumour antigens was described in 1988 in mice (Plaen *et al*, 1988) and 1991 in humans (VanderBruggen *et al*, 1991). Today, it is certain that tumour-specific T-cells can be found in high numbers among tumour-infiltrating lymphocytes (TIL) (Romero *et al*, 1998), but this T-cell response usually does not translate into healing progress. Tumour antigen reactivity usually is weak, these T cells are naturally tolerant of host cancer cells (Pardoll, 2002). Obviously, cancer cells derive from self and appear, in general, more self than non-self to the immune system. Numerous attempts have been made to break this tolerance in order to establish a full-blown T-cell attack against tumour burden, with some success regarding induction of regression but limited success regarding curation (see Jaeger *et al* (2002) for a review on vaccination strategies). It has been suggested that many of these attempts failed because only a particular part of the immune repertoire has been spoiled by these experiments, while a concerted effort is required. For instance, in one advanced experimental setting, dendritic cells (DC) loaded with known melanoma peptide antigens were used to prime T-cell clones from melanoma patients *in vitro*. Growth and clonal expansion of a highly reactive CD8 clone and backtransfer to patients resulted in a dramatic homing of re-injected cells at metastatic sites, but this increased tumour cell recognition by T cells did not propagate into clinical benefit to the patients (Yee *et al*, 2002), since antigen-negative tumour cells were out of target and escaped. Because a combination of CD4 and CD8 responses is required for sustained memory and antitumour activity (Pardoll and Topalian, 1998), a panel of T-cell clones is probably needed to address the genetic and antigenic heterogeneity of tumour cells (Loeb, 2001). Along this line, in another study from 2002, polyclonal T cells were used with greater success. TIL of mixed CD4 and CD8 clonality were used for expansion and re-application to patients, and 6/13 patients experienced partial responses (Dudley *et al*, 2002). The procedure of extracting TILs, priming and expansion *in vitro* and application to the host over longer periods is technically demanding and laborious, thus expense will be a limiting factor for the design of larger clinical trials.

Rather than extracting reactive T-cells from an individual patient, another broadly explored strategy was to define peptide antigens characterising an individual tumour and vaccinating either using the peptide directly or by loading autologous DC with peptide. Only one or a small number of antigens was used in these trials, and again the clinical outcome was limited, with more favourable outcomes for DC-loaded *vs* peptide-alone strategies, but response rates rarely exceeding 30% (Parmiani *et al*, 2002) and uncertain benefit on survival. It should be noted that all tumour antigens used in clinical vaccination trials so far are not canonically tumour specific, but are naturally expressed either in a certain state of differentiation like embryogenesis or are expressed in immunoprivileged sites such as testis or are even expressed at a low level in normal tissue. It may be that it is not one or a few antigens that distinguish tumour from normal tissue, but rather an un-normal composition of antigens. In the latter case, a larger spectrum of antigens – defined or undefined – from a patient's individual disease case must be used for any vaccination strategy. For larger or inoperable tumours, a longer therapy regimen might be advisable, which must address the problem of lesions genetically varying in time, such that antigenic drift has to be considered by regularly re-adjusting the panel of antigens in use. In a recent draft, Rammensee and co-workers outlined such a sophisticated strategy including differential gene expression and HLA analysis comparing an individual patient's normal and tumour tissue using DNA microarrays, bioinformatic data analysis to define potential antigens, on-the-spot clinical grade peptide synthesis of antigens, vaccination and monitoring (Rammensee *et al*, 2002). If antigen drift or genetically distinct cell layers within the tumour are to be considered, this procedure has to be repeated several times during an individual patient's therapy. Again, such a strategy poses formidable technical and logistical challenges; it will be applicable only on a very limited number of patients in the near future. It must be noted that those tumours which lost most of their MHC surface molecules and which can only be recognised by natural killer cells cannot be addressed by this strategy.

In the following, I will assume that in cases of spontaneous regression tumour tolerance has been broken and regression was the result of a full-blown immune attack. I will collect evidence that high fever was a time correlate in many cases of spontaneous regression, and will argue that fever might play a dominant role in helping the immune system to overcome tolerance.

### Fever and spontaneous regression

A correlation between regression and fever was noted long ago, but forgotten later on. Rohdenburg (1918) stated, ‘The greatest number of spontaneous regressions have occurred following incomplete surgical removal of the tumour, next in order of frequency during some acute febrile process…’. Even for the first group he remarks, ‘In some of the histories of this group it is definitely stated that immediately after operation a high temperature developed, which continued without remission for several days’, so he distinguished infectious and postoperative fever. He collected 302 cases of regression, with 77 cases including 44 complete remissions belonging to ‘those reports which stand a most rigid scrutiny’ of correct diagnosis. In 27/302 cases the ‘assigned cause of absorption’ was acute infection, in 69 cases it was ‘incomplete operation’, which, according to Rohdenburg's observation, was often accompanied by high temperature of several days duration. The most frequent infection was erysipelas, an infection causing particularly high fever and producing exotoxins that can function as superantigens, giving rise to polyclonal expansion of T-cells. But also small-pox, pneumonia, malaria and acute tuberculosis were observed. Diamond and Luhby (1951) reported 26 spontaneous remissions in a cohort of 300 cases of childhood leukaemia, 21/26 (80%) were accompanied by infection. Everson and Cole, in their 1966 review, noted in 8/176 cases a severe infection preceding regression (Everson and Cole, 1968; Cole, 1981); however, they probably overlooked some concurrent infections, since Stephenson *et al*, in their 1971 publication, used the same 176 cases and added 48 cases, but found that in 62/224 cases (28%) either an infection or a persistent temperature elevation was reported prior to regression (Stephenson *et al*, 1971). Only vague indication about the involvement of febrile processes is given in the review of Challis and Stam (1990), who state that infection and ‘operative trauma’ were reported in about 4% of the cases. In melanoma, a particular immunogenic variant of cancer, about 1/400 patients experience a spontaneous regression. In a collection of 68 well-documented cases of regression from melanoma, Maurer and Koelmel (1998) found 21 cases with febrile infection preceding regression (31%), in a majority of cases (nine) identified as erysipelas.

The rate of fever concurrent with regression was most likely biased by the awareness of the authors, both of original reports and reviews. Fever, in particular during the second half of the 20th century, which is characterised by heavy use of antibiotics, was regarded unequivocally by main-stream medicine as an unnecessary, weakening state which should be avoided or prevented. The situation has not changed much today. For this reason, if one wants to estimate the rate of fever preceding regressions and remission, one must assume a rate higher than the approximately 30% rate indicated by the Stephenson review, or even much higher for cancers of haematopoetic origin (80%) as indicated by the review of Diamond and Luby, because preceding fever most likely often was not reported. If fever often precedes regressions, there might be a causative connection. This hypothesis can be substantiated by evidence from studies unrelated to spontaneous regression and remission.

### Fever and bacterial extracts

Professor Busch in 1868 introduced the infection of cancer patients by purpose as a novel strategy to treat cancer. He achieved a dramatic regression with his first patient using live *Streptococcus pyogenes* bacteria, the pathogen leading to erysipelas, published in the German Journal ‘Berliner Klinische Wochenschrift’ (Busch, 1868). Beginning in 1891, this strategy was exploited by Coley, who had some reading knowledge of German (Hall, 1998). Coley systematically applied *Streptococcus pyogenes* extracts – later called ‘Coley's toxin’ – to cancer patients and achieved a remarkable rate of regressions. A retrospective compilation of cases considered inoperable at the time of treatment between 1891 and 1936, which was conducted by Wiemann and Starnes (1994, Table 2), determined a remission rate of 64% (108/170) and a 5-year survival rate of larger than 44%. Coley used to inject his extract once or twice a week over a period ranging from a few weeks to several months. His method became quite famous and was tested on hundreds of patients by him and contemporary physicians, but overshadowed by the development of X-ray treatment which was regarded to be much more powerful and of broader applicability. Unfortunately, Coley failed to establish a standard procedure but rather varied the regimen (extract preparation, treatment duration, injection frequency, injection site, dosage) throughout his career, making it difficult to compare his case studies and to pinpoint crucial parameters. His daughter Helen Coley-Nauts re-examined his case studies with respect to differences in bacterial preparation and clinical outcome (Nauts and McLaren, 1990). It is apparent from her publications, but had escaped Coleys attention, that there was a correlation between the potency of a particular extract preparation to induce high fever and rate of curation: 3/13 different preparations had the highest rate of curation (survival longer than 10 years) and were the most powerful in inducing febrile reactions. Also, better clinical response correlated with duration of treatment: if the injections were given over 6 months, an astonishing 80% of inoperable sarcoma of soft tissues survived for 5–88 years (Nauts and McLaren, 1990).

Standardised commercial bacterial extracts similar to Coley's toxin appeared on the market in the 50s and 60s (MBV-mixed bacterial vaccine, Bayer; Vaccineurin, Suedpharma; Picibanil, OK-432, Chugai). Attempts to repeat his successes half a century later using these products were of mixed benefit. In few of these studies particular attention was paid to the induction of high fever possibly reaching 40°C on one side and long treatment over at least several weeks on the other side, both of which appear to correlate with improved outcome (for reviews, see Nauts and McLaren, 1990; Hobohm, 2001). More important, in contrast to Coley's time, most of the patients treated with commercial extracts had prior or concurrent chemotherapeutic treatment with obvious immunocompromising effects, potentially diluting the effect of an immunostimulatory therapy.

### Epidemiology

Since cancer is usually a slowly progressing disease with occasionally long periods of dormancy, putative beneficial fever effects should also precipitate as preventive efficacy. This can indeed be found. In a cohort of 603 melanoma patients compared to 627 population controls, an inverse correlation was found between melanoma risk and number of recorded infections on the one hand and between melanoma risk and fever height on the other hand, leading to a combined reduction of melanoma risk of about 40% for people with a history of three or more infections with high fever above 38.5°C (Koelmel *et al*, 1999). Mastrangelo *et al* (1998) report a striking inverse correlation between the number of infections and mortality from tumours in Italy in the period 1890–1960: every 2% reduction in the number of infectious diseases was followed by a 2% increase in tumours about 10 years later.

### Fever immunology

How could fever act on cancer cells? Fever is used by organisms as diverse as fish, amphibians, reptiles and mammals (see for reference Basu and Srivastava, 2003). Since fever is metabolically expensive, it must provide substantial advantage to the host. Surprisingly little is known about immunological effects mediated by fever, a lack of understanding that might be attributable in part to the common ignorance in clinical practice with respect to benefits fever might provide. Post-operative infections can be prolong survival: patients developing empyema after lung cancer surgery have improved 5-year survival (50% (*n*=18) *vs* 22% (*n*=411)) (Ruckdeschel *et al*, 1972). In this light, it seems unfortunate that fever is usually suppressed in hospital routine.

Effects of hyperthermia, that is, heat applied externally resulting in less and different cytokine production compared to real fever, have been examined to some extent. It has been shown *in vitro* that DC treated with fever-like heat (41°C, 6 h) was significantly superior compared to non-heat-treated DC in stimulating T cells both in the presence or without antigen. Heat treatment of DC activates NF-*κ*B, a transcription factor regulating a number of immunological genes. Similar results were obtained *in vivo* in mice using hyperthermia (41°C, 1 h) as heat treatment (Basu and Srivastava, 2003). Tumour cells are more vulnerable to heat than normal cells and undergo necrosis to a larger extent (Dickson, 1979; Trieb *et al*, 1994). Heat shock protein HSP90 released from cells as a result of necrotic death has been shown to cause maturation of DC (Basu *et al*, 2000). Immunogenic HSP–peptide complexes are displayed to a larger extent on cancer cells after heat treatment at least in some cancers (Udono and Srivastava, 1993; Multhoff *et al*, 1995), an interesting feature since these complexes can activate natural killer (NK) cells (Botzler *et al*, 1996), offering a second defence line independent of MHC-restricted immunogenicity provided by CD4+/CD8+ T cells. Perhaps fever can generate a missing costimulatory signal via DC needed by resting tumour-specific T cells for full activation, followed by partial or complete ‘spontaneous’ regression in an established tumour, to eradication of dormant cancer cells in a young tumour (prevention) or to eradication of residual cancer cells after surgery (improved survival after postoperative infection). The involvement of adjuvants should provide additional therapeutic options, as might strategies to block inhibitory pathways of T-cell activation temporarily (Leach *et al*, 1996; Rubinfeld *et al*, 1997).

Since there is much evidence that fever alone can have beneficial effects, experiments using pyrogenic cytokines for treatment of cancer (e.g. interleukin (IL)-1, IL-6, tumour necrosis factor (TNF), ciliary neurotropic factor (CNTF), interferon-(IFN)-alpha) should discuss cytokine and fever effects separately.

Clinically controlled induction of fever as part of therapy regimen should be re-considered, now carefully addressing fever height, fever duration, fever schedule and distance in time between fever and chemotherapy, before or after.

## CONCLUSION

A large fraction of spontaneous regressions and remissions described in the literature was preceded by a hefty feverish infection. The hypothesis that fever can have therapeutic value can be brought in line both with successful historical attempts to apply fever using bacterial extracts and with immunological evidence. Putative beneficial effects of fever should as well act preventive, and indeed epidemiological studies show that a personal history of feverish infections reduces the likelihood to develop cancer later.

Immunological strategies to break cancer cell tolerance should be individualised rather than generic. Fever induction will necessarily induce an individual response, which even addresses the problem of antigenic drift in time. Today we should be able to induce and control fever much better than 100 years ago. The discussion should be re-opened if and how to best scrutinise fever therapy in the future (see http://bioinfo.tg.fh-giessen.d e/fever-and-cancer for online discussion).

### Conflict of interest

No financial or personal relationships between the author and other people or organisations exist that could inappropriately influence this work.
